# Rapid culture-based diagnosis of pulmonary tuberculosis in developed and developing countries

**DOI:** 10.3389/fmicb.2015.01184

**Published:** 2015-11-03

**Authors:** Shady Asmar, Michel Drancourt

**Affiliations:** Faculté de Médecine, URMITE, UM63, Centre National de la Recherche Scientifique 7278, IRD 198, Institut National de la Santé et de la Recherche Médicale 1095, Aix Marseille UniversitéMarseille, France

**Keywords:** culture media, *Mycobacterium tuberculosis*, diagnosis, developing countries, pulmonary tuberculosis

## Abstract

Culturing *Mycobacterium tuberculosis* remains the gold standard for the laboratory diagnosis of pulmonary tuberculosis, with 9 million new cases and 1.5 million deaths mainly in developing countries. Reviewing data reported over 20 years yields a state-of-the-art procedure for the routine culture of *M. tuberculosis* in both developed and developing countries. Useful specimens include sputum, induced sputum, and stools collected in quaternary ammonium preservative-containing sterile cans. The usefulness of other non-invasive specimens remains to be evaluated. Specimens can be collected in a diagnosis kit also containing sampling materials, instructions, laboratory requests, and informed consent. Automated direct LED fluorescence microscopy after auramine staining precedes inoculation of an egg-lecithin-containing culture solid medium under microaerophilic atmosphere, inverted microscope reading or scanning video-imaging detection of colonies and colonies identification by recent molecular methods. This procedure should result in a diagnosis of pulmonary tuberculosis as fast as 5 days. It may be implemented in both developed and developing countries with automated steps replaceable by manual steps depending on local resources.

## Introduction

In 2013, the World Health Organization reported nine million new cases of tuberculosis worldwide and 1.5 million deaths (WHO, 2014). Pulmonary infection is the most prevalent contagious clinical form of tuberculosis, and is therefore of prime public health importance (Saranchuk et al., 2007). Commercially available, confined, real-time PCR kits contribute to rapid DNA-based detection of the causative agent *Mycobacterium tuberculosis* (Steingart et al., 2013). DNA-based techniques can detect dead *M. tuberculosis* organisms without any medical significance and are also less sensitive than culture (Bicmen et al., 2011). Therefore, culturing *M. tuberculosis* remains the gold standard for the laboratory diagnosis of tuberculosis (Tenover et al., 1993; Rageade et al., 2014). Culture still relies on a relatively cumbersome and lengthy process starting with the collection of appropriate clinical specimens and their transport to the laboratory, decontamination of clinical specimens likely to be contaminated by a commensal flora, inoculation and incubation of appropriate media, growth detection and mycobacteria identification. However, recent advances in each one of these steps significantly decreased the delay for culture-based detection of *M. tuberculosis*.

In this article we review the existing literature related to each one of these steps in order to promote a state-of-the-art protocol for the rapid, routine culture-based diagnosis of pulmonary tuberculosis.

### Search strategy and selection criteria

References for this review were identified through searches of PubMed for articles published from January 1990 to March 2015, by the use of the terms Ziehl–Neelsen, auramine, fluorescence, decontamination, clinical specimen, culture, *Mycobacterium tuberculosis*, and tuberculosis, either alone and combined. Additional relevant articles published between 1918 and 1990 were identified through searches in the authors' personal files, in Google Scholar and the Springer Online Archives Collection. Articles resulting from these searches and relevant references cited in those articles were reviewed. Articles published in English and French were included (Datasheets 1, 2).

### Clinical specimens

Sputum specimens obviously have to be collected for the diagnosis of pulmonary tuberculosis (Koch, 1882). In children, HIV-infected patients and elderly patients who do not expectorate, induced sputum is obtained using nebulizined, warmed 3–20% sodium chloride solution (Gonzalez-Angulo et al., 2012) with a 76.4–100% success (Hepple et al., 2012) and a culture sensitivity of 63–96.6% superior to that of gastric wash (Zar et al., 2000, 2003, 2005; Brown et al., 2007; Morse et al., 2008; Bell et al., 2009; Moore et al., 2011; Ruiz Jiménez et al., 2013; Table S1). Nasopharyngeal aspirate may also induce sputum but its culture is less sensitive than that of gastric wash and induced sputum (Franchi et al., 1998; Owens et al., 2007). Laryngeal swab has an average higher culture sensitivity than gastric wash (Mankiewicz, 1953; Lloyd, 1968; Bhandari et al., 1971; Thakur et al., 1999; Oberhelman et al., 2010) and saliva is an alternative specimen (González Mediero et al., 2014). Both specimens require additional investigations to confirm their usefulness for the diagnosis. In patients with neurological disorders including comatose patients and other non-expectoring patients, swallowed acid-resistant *M. tuberculosis* can be cultured in the gastric wash. This uncomfortable and harmful procedure exhibits disappointing 23–72.2% sensitivity (Table S1). We showed that *M. tuberculosis* has been isolated from gastric wash specimens with 60% sensitivity, not different from the 64% sensitivity observed in the stools of the same patients (Bonnave et al., 2013). Contrary to previous studies (Donald et al., 1996), we found 54.2–64% stool culture sensitivity (El Khéchine et al., 2009; Bonnave et al., 2013) and are routinely culturing stools for the diagnosis of pulmonary tuberculosis. We propose that all invasive procedures, including gastric wash (Vandal et al., 2009; Cruz et al., 2013), string test (Vargas et al., 2005), bronchoalveolar wash (Norrman et al., 1988), and biopsies should not be routinely used but reserved to cases where non-invasive procedures have failed.

### Transport and storage of specimens

Storage of specimens at −20°C preserves 100% viability of *M. tuberculosis* (Tessema et al., 2011), storage at 2–4°C preserves 94.4% viability (Lumb et al., 2006; Palomino, 2007) but room temperature storage decreases viability by 3–4 log and increases overgrowth of contaminants by 1–1.5 log (Paramasivan et al., 1983). Therefore, for room temperature storage of >4 h, a preservative must be used (Pardini et al., 2005). Quaternary ammonium compounds cetylpyridinium chloride (CPC, 1%; Selvakumar and Narayana, 1993; Rieder and Rieder, 1998), cetylpyridinium bromide (CPB, 0.6%; Smithwick et al., 1975), and chlorehexidine gluconate (CHX, 1 and 0.7%; Peres et al., 1988; Asmar and Drancourt, 2015) are routinely used. CHX yields 1–2 log more mycobacteria than NALC–NaOH and NALC–NaOH–Oxa (Ferroni et al., 2006; Asmar and Drancourt, 2015) and effectively decontaminates stools (Tessema et al., 2011) and sputum (Asmar and Drancourt, 2015). We routinely use CHX for rapid, simple and inexpensive preservation and decontamination of specimens as CHX can be stored at room temperature for a year. Quaternary ammonium-treated specimens should not be frozen (Smithwick et al., 1975; World Health Organization, 2003; Sankar et al., 2009), should not be further decontaminated and must be neutralized by an egg-based solution or by Bacto neutralizing buffer (Master, 1992) and inoculated on a medium containing egg lecithin (Smithwick et al., 1975). Culture yield is significantly higher in quaternary ammonium-preserved specimens than in untreated specimens (Selvakumar and Narayana, 1993; Selvakumar et al., 1995; Bobadilla-del-Valle et al., 2003; Pardini et al., 2005; Pal et al., 2009). Alternatively, 18–24-h incubation in trisodium phosphate (Jena and Panda, 1999) ensures a proper decontamination (Chauhan, 1999; Jena and Panda, 1999, 2004).

### Pulmonary tuberculosis diagnosis kit

We used a “tuberculosis kit” to standardize specimen collection and their manipulation in the laboratory. The kit features three 60-mL sterile containers for sputum samples and three 180-mL sterile containers for stool samples, which are labeled with the patient's number, patient's name, date of birth, the ward number, sample type, date of sampling, and a code bar. An inquiry sheet will filled out by the doctor in charge and a specimen sheet filled out by the nurse for each specimen listing in addition to the samples' label information, the lab destination, the patient's temperature at the sampling time, the patient's ongoing treatment, if any, and an instruction sheet.

### Decontamination of specimens

Unpreserved specimens can be decontaminated and homogenized using the N-acetyl-cystein 2% NaOH method (Kent et al., 1985), derived from Petroff's method (Petroff, 1915), which is compatible with all culture media and PCR-based analyses (Zingué et al., 2013). Limitations include the centrifugation step and 24-h preemption (Burdz et al., 2003). Alternatively, it was shown that hydrochloric acid-based decontamination (Kent et al., 1985) was less effective than sodium lauryl-sulfate or NALC–NaOH (Langerová and Tacquet, 1968). Likewise, 3%-sulfuric acid decontamination yielded more non-tuberculous mycobacteria (NTM) but less *M. tuberculosis* isolates than NALC–NaOH (*p* = 0.39; Buijtels and Petit, 2005). Oxalic acid (ethanedioic acid, 5%) yields results similar to sodium lauryl sulfate, NALC–NaOH and 6%-sulfuric acid (Pathak and Deshmukh, 1973) which can be used for heavily contaminated specimens, including *Pseudomonas* species (Pfyffer et al., 2003). Sodium lauryl sulfate decontamination is effective (Langerová and Tacquet, 1968) but treated specimens should not be inoculated in liquid medium (Pfyffer et al., 1997) and are inappropriate for PCR-based assays (Zingué et al., 2013). Therefore, sodium lauryl sulfate is no longer used for decontamination.

Hypertonic saline–sodium hydroxide (HS–SH; Ricaldi and Guerra, 2008) mixing 1 mL sputum with 1 mL of 7% saline as a mucolytic agent and 1 mL 4% sodium hydroxide (NaOH), significantly reduces contamination but does not increase the yield of *M. tuberculosis* (Ganoza et al., 2008; Ricaldi and Guerra, 2008; Chaudhary and Mishra, 2013). This method is less expensive and simpler than the standard NALC–NaOH method; the NaCl solution can be stored at room temperature for several days in contrast to the NALC solution. The resulted sediments can be used in the different biomolecular analyses and can be preserved for later use for drug sensitivity (Zingué et al., 2013). For this reason, this method is a promising candidate for evaluation and adoption by national TB control programs in developing countries (Ricaldi and Guerra, 2008).

### Microscopic examination of specimens

**Figure 1:**
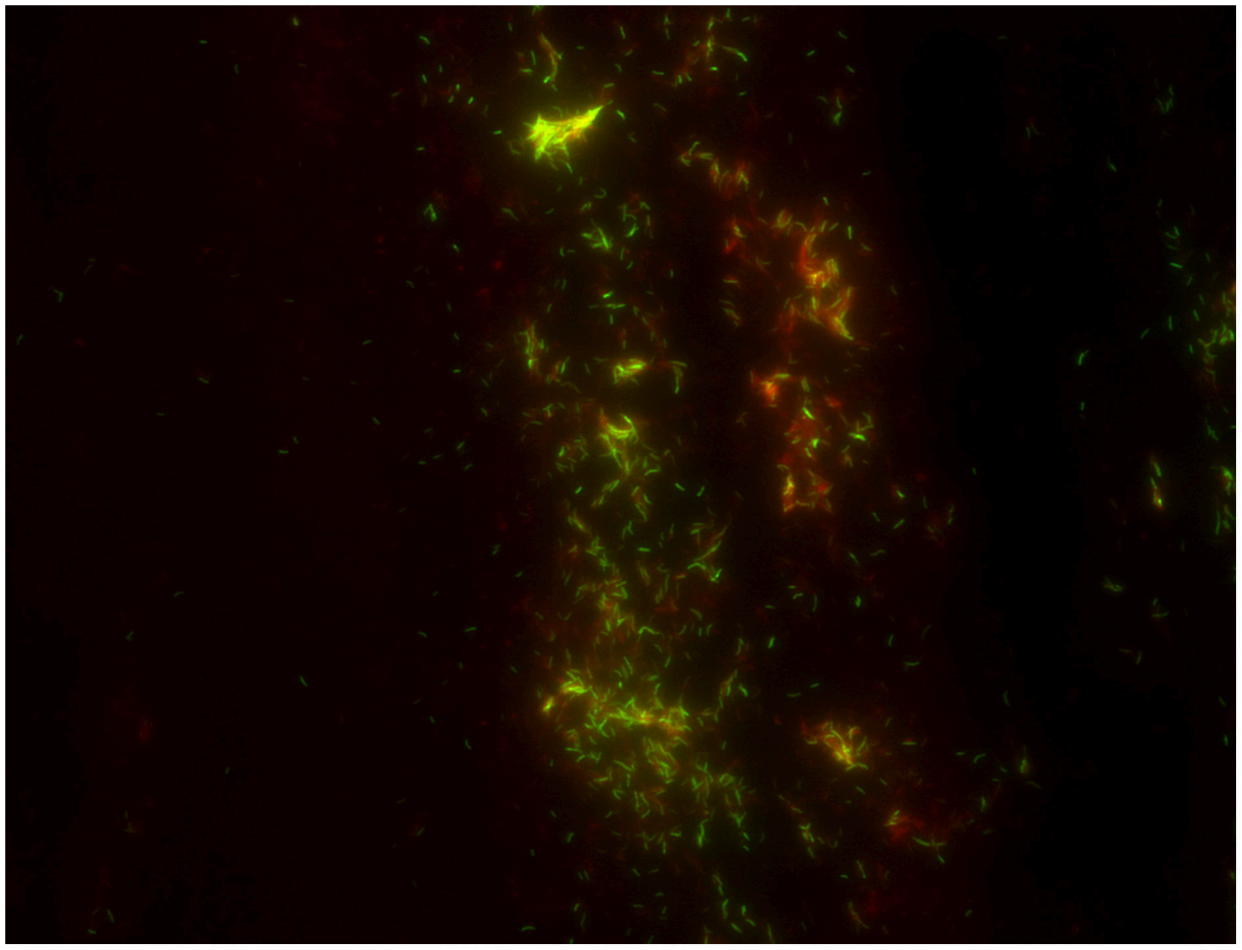
*****M. tuberculosis*** stained by fluorescence auramine–rhodamine**.

The cost-effectiveness of direct microscopic examination for the rapid diagnosis of pulmonary tuberculosis remains to be demonstrated in the perspective of new, alternative direct diagnosis techniques. Indeed, extending the use of real-time PCR has a higher cost than direct microscopic examination (0.57$ for microscopy after Ziehl–Neelsen staining vs. 19.56$ for PCR) but is probably more cost-effective when treatment costs are included (412 for microscopy after Ziehl–Neelsen staining vs. 382 for PCR) and yields a clear performance superiority (Table S2). Nevertheless, direct microscopic examination remains a standard step in the laboratory diagnosis of TB. A few studies indicated that concentration of *M. tuberculosis* by using magnetic beads increased the yield of microscopic examination (Liu et al., 2013; Wang et al., 2013), however this expensive method has not been widely used. Staining methods for *M. tuberculosis* rely on the acid-resistance of its lipid-rich cell wall (Liu et al., 1996). The “hot-staining” Ziehl–Neelsen method remains the most widely used screening method (Selvakumar et al., 2002) while carbol-fuchsine “cold-staining” methods eliminating the heating step (Kinyoun, 1915; Tan Thiam Hok, 1958; Gokhale, 2001) are safer and more practical. The relative advantages are disputed (Engbaek et al., 1969; Vasanthakumari et al., 1987; Somoskövi et al., 2000; Van Deun et al., 2005) but WHO and the International Union against Tuberculosis and Lung Disease IUATLD advocate the Ziehl–Neelsen hot-staining technique using a 0.3% concentration of basic fuchsine (Weyer, 1998; International Union against Tuberculosis and Lung Disease, 2000). Observations made at X 1000 magnification yielded variable low sensitivity of 22–80% compared to culture (Kaufmann and Hahn, 2003). This is particularly true in children (Getahun et al., 2007) and HIV-co-infected patients (Swai et al., 2011). Alternatively, *M. tuberculosis* can be stained in bright yellow-orange by fluorescent auramine O or auramine–rhodamine (Figure 1; Weiser et al., 1966). Observations are made at X 250 using fluorescent light-emitting diode microscopy (LED-FM) which can be operated on batteries for field use (Anthony et al., 2006; World Health Organization, 2011; Marzouk et al., 2013). Compared to Ziehl–Neelsen staining, LED-FM reduces observation time by 50–75% (Ba and Rieder, 1999; Marais et al., 2008; Habtamu et al., 2012; Table S3), increases sensitivity (Table S4; Steingart et al., 2006; Marais et al., 2008; Trusov et al., 2009; World Health Organization, 2011; Marzouk et al., 2013) for an estimated 1.97 ± 0.71$ cost lower than the 2.20 ± 0.58$ cost of Ziehl-Neelsen staining (Xia et al., 2013). LED-FM detection of esterase activity specific for viable *M. tuberculosis* cells (Hamid Salim et al., 2006; Van Deun et al., 2012; Datta et al., 2015) unfortunately had limited specificity and sensitivity (Lawn and Nicol, 2015). Promisingly, several computational algorithms recognize Ziehl–Neelsen stained acid-fast bacilli in digitalized images. An automated multi-stage, color-based Bayesian segmentation discriminates confirmed AFB cells (green) from possible AFB cells (blue) from artifacts (red; Sadaphal et al., 2008) by using a neural network (Siena et al., 2012) or methods based on Otsu thresholding and k-means clustering approach (Rachna and Mallikarjuna Swamy, 2013). Theoretically these algorithms have 88–98% accuracy (Siena et al., 2012; Rachna and Mallikarjuna Swamy, 2013) but routine evaluations are lacking. The TBDx system (Signature Mapping Medical Sciences, Herndon, USA) automatically loads slides onto a microscope, focuses and digitally captures images to classify auramine-stained smears as positive or negative using computerized algorithms. It routinely showed a significant higher sensitivity than manual microscopy (75.8 vs. 52.8%) but a lower specificity of 43.5 vs. 98.6% (Lewis et al., 2012). Automated reading systems may require additional work or further manual microscopy of positive specimens in order to improve specificity.

### Culture media

Decontaminated specimens can be inoculated in solid and liquid media with WHO advocating parallel inoculation in both media in order to combine the higher specificity of solid media with the higher sensitivity of liquid media (Nolte and Metchock, 1999). Incubation at 37°C is optimal and we showed that *M. tuberculosis* is a microaerophilic organism and took this characteristic into account to develop new culture media (Ghodbane et al., 2014; Asmar et al., submitted). A 5–10% CO_2_ enrichment of atmosphere stimulates primary isolation (Gottlieb et al., 1964). In 1882, the seminal culture of *M. tuberculosis* was achieved for seven sputum specimens within 1 week using a coagulated bovine serum solid culture medium (Koch, 1882; Cambau and Drancourt, 2014). Further formulations included the incorporation of 5% glycerol (Nocard, 1887), the combination of potato and glycerol and a blood-based medium supplemented with glucose and glycerol (Bezançon and Griffon, 1903a). In 1903, the first egg-yolk agar medium (Bezançon and Griffon, 1903b) was reported, further complemented by glycerol (Lowenstein, 1931) and malachite green to inhibit contaminants (Jensen, 1932). This Lowenstein–Jensen (LJ) medium is solidified by coagulation at 83°C for 40 min. It remains the most commonly used culture medium worldwide. Coletsos complemented the LJ medium with sodium pyruvate, gelatin, sodium glutamate, sodium pyruvate, activated carbon and oligonucleotides and lowered the concentration of glycerol (Coletsos, 1960). These modifications optimized the isolation of *Mycobacterium bovis, Mycobacterium africanum*, and mycobacteria from treated patients. In both media, eggs buffer harmful effects of quaternary ammonium compounds against *M. tuberculosis*. The advantages of these inexpensive media are high sensitivity and the characteristic morphology of colonies. However, they are sensitive to the quality of organic compounds (eggs), long to prepare and rapidly perishable.

The Middlebrook 7H10 medium contains mineral salts, glucose, albumin bovine (V fraction), amino acids, oleic acids and sodium pyruvate. The Middlebrook 7H11 medium was supplemented with enzymatic digest of casein providing nitrogen, vitamins and amino acids (Cohn et al., 1968). Their transparency facilitates early detection of flat, dry, rough and brittle-looking *M. tuberculosis* colonies. Due to its high cost, Middlebrook media are rarely used routinely in countries with limited resources. Thin layer agar (TLA) is a solid medium-based test, which, on a similar principle as the Microscopic Observation Drug Susceptibility assay (MODS) method (Lazarus et al., 2012), relies on the inoculation on a thin layer of Middlebrook 7H11 agar for the detection of cording *M. tuberculosis* microcolonies using an inverted microscope (Runyon, 1970). Later, incidental isolation of *M. tuberculosis* on blood agar (Drancourt and Raoult, 2007) suggested that this routine medium could support isolation of *M. tuberculosis*. (Drancourt et al., 2003; Drancourt and Raoult, 2007; Coban et al., 2011) We further modified its formulation to obtain a MOD4 medium (Ghodbane et al., 2014) and we recently developed a blood-free, serum enriched MOD9 medium which supported isolation of *M. tuberculosis* in 25 h (Ghodbane et al., 2015). Later media contained an optimized concentration of ascorbic acid as an antioxidant in order to mimic a microaerophilic atmosphere for the optimal growth of *M. tuberculosis* in ambient air or in a 5% CO_2_-enriched atmosphere (Ghodbane et al., 2014; Asmar et al., submitted). Solid culture media ease the detection of contaminants, allowing to pick up the right colony for further analyses. Moreover, the morphology of colonies facilitates differentiation between *Mycobacterium* species in mixed infections (Jun et al., 2009).

Among liquid media, the Dubos broth (Dubos et al., 1950) contains inorganic salts, enzymatic digest of caseine and acid L-asparagine as a source of nutrient, polysorbate 80 and oleic acid ester as a source of essential fatty acids and bovine albumin as a protective agent from the binding of free fatty acids toxic to mycobacteria. The Middlebrook 7H9 broth designed in 1955, contains inorganic salts, sodium citrate to hold certain inorganic cations in solution and albumin. It may be supplemented with glycerol, growth-promoting polysorbate 80 and OADC solution (Oleic acid, Bovine Albumin fraction v, Dextrose as an energy source and detoxifying Catalase). Middlebrook 7H12 broth derives from Middlebrook 7H9 broth by supplementation with glycerol, bovine serum albumin, caseinehydrolysate, catalase and ^14^C-Palmitic acid as a marked source of carbon (Middlebrook et al., 1977). Modified versions of these media are incorporated into different automated systems. Biphasic media combine LJ slant with a liquid culture medium, and a colorimetric indicator (Cui et al., 2012); or a Middlebrook 7H11 slant and a Middlebrook 7H9 broth (Ghatole et al., 2005). In particular, the SEPTI-CHEK AFB (Becton Dickinson, Cockeysville, USA) consists of 20 mL of modified Middlebrook 7H9 in a CO_2_-enriched atmosphere (SEPTI-CHEKAFB Mycobacteria Culture Bottles), supplemented by a mixture of enrichment supplement and antibiotics cocktail with a SEPTI-CHEK AFB Slide attached to the bottle after inoculation. The SEPTI-CHEK AFB Slide contains a Middlebrook 7H11 agar, a Middlebrook 7H11 medium supplemented with malachite green and egg powder and a chocolate agar on each one of its three sides. The first two sides are intended for growing mycobacteria and the third side for growing contaminants.

### Growth detection

Detecting growth has been key to significant progress over the past decades. In solid media, naked-eye detection of colonies can be helped in the TLA by using an inverted microscope or by using an autofluorescence detector in order to detect fluorescence light emitted by *M. tuberculosis* micro-colonies when excited by a 450–550 nm wavelength range (Ghodbane et al., 2014). Alternatively, the MB redox (Biotest, Dreiech, Germany) incorporates tetrazolium salt as a redox indicator in the Kirchner medium. Mycobacteria reduce colorless tetrazolium salt in pink-violet insoluble formazan visible by naked eye. Tetrazolium salt, however, is toxic for *M. tuberculosis* (Ghodbane and Drancourt, unpublished data). MODS is a liquid medium-based test combining *M. tuberculosis* growth detection and drug susceptibility assay directly from sputum samples based on the property of growing *M. tuberculosis* to form cords. A decontaminated pellet suspended in a modified 7H9 based broth supplemented with OADC and antibiotics is placed in a sealed 24-well plate, with wells containing a negative control without antibiotics, or isoniazid or rifampicin for the drug susceptibility assay. The characteristic cording growth of *M. tuberculosis* is detected after 5-day incubation at 37°C using an inverted light microscope (Lazarus et al., 2012).

In liquid medium, colonies are not observed and growth is deduced from the variations of a physical parameter. These measures are indirect proxies for growth in contrast with the direct visualization of colonies on solid medium. In the Biorad Bio FM (BioRad, Hercules, USA), growth detection relies on naked-eye observation of blue-violet colored grains. In the Bactec MGIT 960® (Becton Dickinson, East Rutherford, USA) growth is inferred from decreasing oxygen tension in a tube detected by a ruthenium pentahydrate oxygen sensor embedded in silicon at the bottom of the tube, fluorescing under UV light (Tortoli et al., 1999). In the BacT/Alert MB® (bioMérieux, Craponne, France), growth is inferred from increasing CO_2_ tension in a bottle. The Versa Trek® machine (TREK Diagnostic Systems, Oakwood Village, USA) uses small glass bottles containing a cellulose sponge to increase contact surface with a modified Middlebrook 7H9 broth; growth is inferred from the decreasing pressure in the bottle monitored by a nanometer.

Reviewing data published for 15 years (Tables S5, S6) indicates that automated culture systems have a higher sensitivity and a shorter detection time compared to conventional solid culture protocols. The Bactec 460 TB has the highest sensitivity and the lowest contamination rate when compared to Bactec MGIT 960, BacT Alert 3D and MB redox) and LJ and Coletsos media and the second lower detection time (after Bactec MGIT 960). Bactec MGIT 960 is the fastest culture system when compared to the other culture systems and media, a clearly higher sensitivity when compared to the conventional used solid media (LJ, Coletsos) but a non-conclusive superiority when compared to other culture systems (BacT Alert 3D, MB redox); the results were variable from one study to another when comparing its contamination ratio to other systems and media (BacT Alert 3D, LJ) and thus non-conclusive.

As for microscopy-based culture detection, the thin layered agar TLA-7H10 showed a 9–19 days reduction with respect to LJ but a higher contamination rate; its sensitivity was variable, higher than LJ in some studies (Gil-Setas et al., 2004; Robledo et al., 2006; Martin et al., 2009) but not in others (Mejia et al., 1999; Idigoras et al., 2000). TLA has a lower sensitivity and longer time to detection than the Bactec MGIT 960 (Supplementary Material: Tables S5, S7). MODS has a significantly higher sensitivity and lower detection time than conventional LJ medium and a similar contamination ratio (Tables S5, S7). Comparing MODS to Bactec MGIT 960 showed no significant difference in sensitivity and a significant rapidity of MODS (Dang et al., 2012; Makamure et al., 2013). The use of “Mycobacterial reporter Fluorophage” is another microscopy-based detection method of *M. tuberculosis* (Jain et al., 2011). The recent advances in this field has allowed the development of high-intensity fluorophages capable to detect *M. tuberculosis* in clinical sputum specimens and to provide drug susceptibility test results in a matter of 12–36 h (Jain et al., 2012). TK medium is a new culture medium that contains different dyes that change the color of the medium depending on the bacteria growing on it. The change of color occurs before the appearance of the colonies. The medium which is initially red turns yellow when mycobacteria grow and green when contaminant bacteria and fungi grow. Many studies proved that TK medium detected mycobacteria in clinical specimens faster than LJ (9.6–16 days for TK vs. 25–29.6 days for LJ; Ercis et al., 2002; Bicmen et al., 2003; Baylan et al., 2004; Altindis et al., 2011; Kocagöz et al., 2012), however it is still slower than Bactec MGIT 960 (5–8.3; Kocagoz et al., 2000; Ercis et al., 2002) and Bactec 460 TB (11.7–12.5 days; Baylan et al., 2004; Altindis et al., 2011). As for the sensitivity and the contamination ratio, the results were variable. In some studies, the TK presented a lower sensitivity for the isolation of mycobacteria and a higher contamination rate than LJ and Bactec 460 TB (Altindis et al., 2011; Baylan et al., 2004), while in another study, the TK medium presented a higher sensitivity and a lower contamination rate than LJ (96.3%, 0.5% for TK vs. 89.3%, 5.33% for LJ; Kocagöz et al., 2012; Table S5). However, a yellow color of the medium is not specific as some streptococci also metabolite the reactive. Moreover, TK medium is pH sensitive and it is necessary to adjust the pH to ~7.4 ± 0.2 after the decontamination step.

The new MOD9 medium we recently developed is promising but still under evaluation (Asmar et al., submitted). This solid culture medium improves the performances of the MOD4 medium, a blood-based medium that allowed the detection of *M. tuberculosis* colonies in 8.4 ± 3 days compared to the 12.55 ± 4.6 days by Bactec MGIT 960; further reduced to 4.75 ± 1.3 days using an autofluorescence detector (Ghodbane et al., 2014). In MOD9 medium, blood has been eliminated. Comparison of MOD9 with LJ (chlorhexidine-0.7% decontamination) showed that MOD9 had a higher sensitivity, shorter time to detection of *M. tuberculosis* and lower contamination rate (95%, 10.5 ± 4.2 days and 1.6%) than LJ (80%, 18.1 ± 6.3 days and 4.4%; Asmar et al., submitted). Furthermore, the comparison of MOD9 medium (chlorhexidine-0.7% decontamination) with Bactec 960 MGIT (NALC–NaOH decontamination) showed that MOD9-chlorhexidine-0.7% had a higher sensitivity, shorter time to detection and a lower contamination rate (95.8%, 10.1 ± 3.9 days and 1.7%, respectively) than Bactec MGIT 960–NALC–NaOH (40.8%, 14.7 ± 7.3 days and 5.7%; Asmar et al., 2015). Combining MOD9 with scanning technology (Advencis-Bio-system) enabled the detection of *M. tuberculosis* colonies in 3.4 ± 2 days vs. 15 ± 6 for Bactec MGIT 960 (Ghodbane et al., 2014, 2015; Table 1).

**Table 1:** **Comparison of sensitivity, contamination rate, time to detection, or isolation of ***Mycobacterium tuberculosis***, advantages and limitations for different culture media or systems**.

**Medium/System**	**Number of studies**	**Sensitivity (%)**		**Contamination rate (%)**		**Time to detection of** ***Mycobacterium tuberculosis*** **(days)**						**Advantages**	**Limitations**
						**Smear positive and negative**		**Smear positive**		**Smear negative**			
		**Range**	**Mean**	**Range**	**Mean**	**Range**	**Mean**	**Range**	**Mean**	**Range**	**Mean**		
Lowenstein–Jensen	62	35.7–98.2	74	0.6–22.1	7.27	11–38	25.5	14–30	25	23–42.2	29.4	Low cost Characteristic appearance of colonies	Slow High contamination rate Dependence in the compounds (egg) quality Long and hard preparation method Short term conservation
Bactec MGIT 960	35	71.1–100	89.4	0.3–23.8	9.3	5–32.8	12.8	7–15.3	11.25	13.4–22.4	16.6	Fast High sensitivity Automated reading High throughput capacity	High cost High hazard risk (centrifugation) High contamination rate Technical expertise Frequent machine maintainance
Bactec 460 TB	24	78.8–100	92.6	0–11.7	4	11.7–18.5	14.4	8–13.8	11.3	16–21.3	18.9	Fast High sensitivity	High cost High hazard risk (centrifugation) Radioactive waste Technical expertise
MB/BacT	14	80–100	91.6	1.8–10.2	5.9	13.8–20.1	16.6	11.3–15.1	12.5	19.6–26.7	21.6	High sensitivity Automated reading High throughput capacity	High cost High hazard risk (centrifugation) High contamination rate No further advantages than other automated systems (Bactec) Technical expertise Frequent machine maintainance
MB Redox	7	72.3–95	84	2.1–8.6	3.6	16–23.6	18.7	6.9–15.9	12.5	15.5–23.8	20.9	Easy read of results (color indicator)	High cost High hazard risk (centrifugation)
TLA	7	67.9–92.6	81.2	1–26	10.1	10.9–14.5	12.8	8.9–10.1	9.8	14.6–20.7	17.5	Low cost Characteristic appearance of colonies	Use of microscopic tools Low sensitivity High contamination rate Cumbersome
MGIT manual	7	63.6–94.4	84.7	1.5–29.3	9.5	10–17.5	13.7	7.2–8	7.6	15–19.1	17	Fast	High cost High contamination rate High hazard risk (centrifugation)
Bactec 9000	3	92.8–100	96.4	7		13.2–26.6	19.9					Fast High sensitivity Automated reading	High cost High contamination rate
Versa TREK/ESP II	2	73.8–84.8	79.3	4.2–18.9	11.5	18.7–19.8	19.3	15.1		15.1		Automated reading High throughput capacity	High cost High contamination rate No further advantages than other automated systems (Bactec) Frequent machine maintainance Cumbersome
MODS	8	66–96	87.6	0.6–7	3.35	7–24	10.9					Fast Low cost High sensitivity	Use of microscopic tools High hazard risk (centrifugation) Cumbersome
TK/DioTK	6	73.7–96.3	85	0.5–3.8	2	9.6–16	13.4					Easy read of results (color indicator)	Under evaluation
Middlebrook 7H11	3	53.4-87.7	67.3	2.7-11	6.85	19.2–31.1	25.1	16.5–17.6	17	21.6–22.3	22	Low cost	Slow Low sensitivity High contamination rate
Coletsos	2	69.9–75.1	72.5	7–16.1	11.5	22.7–26.2	24.5					Low cost Characteristic appearance of colonies	Slow High contamination rate Dependence in the compounds (egg) quality Long and hard preparation method Short term conservation
Bio FM	1	92.3		1.8		12.4						Easy read of results (color indicator)	High cost High hazard risk (centrifugation)
MOD9	2	95–95.8	95.4	1.6–1.7	1.6	10.1–10.5	10.3	6.5–6.8	6.6	12.8–13.4		Faster medium Low cost High sensitivity Characteristic appearance of colonies	Under evaluation
MOD9/Advencis	1	81.2		4		3.4						Faster system High sensitivity Characteristic appearance of colonies Automated reading	High cost Under evaluation

## Conclusions: Integrating culture-based diagnosis of pulmonary tuberculosis in developing and developed countries

As usual in medical microbiology (Lagier et al., 2015), the culture of *M. tuberculosis* must remain the ultimate goal for microbiologists for the diagnosis of pulmonary tuberculosis in order to diagnose live vs. dead organisms, describe the complexity of mixed infections and allow advanced “omics” studies. Current state-of-the art techniques already allow for culture-based diagnosis in less than 48 h in optimal conditions (Ghodbane et al., 2015). The integrated procedure starts with a “Pulmonary Tuberculosis Kit” to standardize the collection of sputum or induced sputum and stools into sterile cans containing 0.7% chlorhexidine. New formats are under development to fit with the point-of-care collection of the kit. Where cost-effective, naked-eye or automated microscopic examination should follow auramine staining and LED-FM and decontaminated specimens should be inoculated onto any egg-lecithin-containing, modified Middlebrook solid culture medium. Rapid microcolony detection may be done in the thin-layer format and binocular microscope or by using scanning video imaging. Identification should be done by MALDI-TOF-MS.

### Conflict of interest statement

Michel Drancourt is co-inventor of MOD5 medium. Shady Asmar and Michel Drancourt are co-inventors of patented MOD9 medium. The authors declare that the research was conducted in the absence of any commercial or financial relationships that could be construed as a potential conflict of interest.

## Supplementary material

Table S1 **Comparison of culture yield or sensitivity for ***Mycobacterium tuberculosis*** isolation from different specimens type**.

Table S2 **Comparison of sensitivity for ***M. tuberculosis*** detection in clinical specimens for molecular technique (PCR) and Microscopic examination techniques (Ziehl-Neelsen and Fluorescence)**.

Table S3 **Comparison of observation time required for each specimen for carbol-fuchsine and fluorescence staining methods**.

Table S4 **Comparison of ***M. tuberculosis*** detection yield or sensitivity in clinical specimens between different microscopic examination techniques**.

Table S5 **Comparison of culture yield, contamination rate and time to detection or isolation of mycobacteria for different culture media or systems**.

Table S6 **Comparison of culture yield, contamination rate and time to detection or isolation of mycobacteria for different culture media or systems excluding microscopy-based system (TLA and MODS) and non-commercialized media (TK)**.

Table S7 **Comparison of the culture yield, the contamination rate and the time to detection or isolation of mycobacteria of microscopic-based culture system (TLA and MODS), TK/Dio TK medium to others culture media or systems**.
