# Supplementary material for: Rapid culture-based diagnosis of pulmonary tuberculosis in developed and developing countries
Source: Front Microbiol. 2015 Nov 3;6:1184. doi: 10.3389/fmicb.2015.01184 (PMC4630581; doi:10.3389/fmicb.2015.01184)
Supplement: Supplementary file 2 [file DataSheet2.PDF]

Reference list (Supplementary data)

1. Abadco D.L., Steiner P. (1992). Gastric lavage is better than bronchoalveolar lavage for isolation of Mycobacterium tuberculosis in childhood pulmonary tuberculosis. *Pediatr Infect Dis J*. 11(9), 735-8.
2. Abe C., Hirano K., Wada M., Tsubura E., Yamanaka M., Aoyagi T., Osumi M., Takeda M., Kurashima A., Yoneyama A., Okuzumi K. (1999). Comparison of the newly developed MB redox system with mycobacteria growth indicator tube (MGIT) and 2% Ogawa egg media for recovery of mycobacteria in clinical specimens. *Kekkaku*. 74(10), 707-13.
3. Ahmed K.S., Raj A., Ahi J.D., Tsalla T. (2013). Diagnosis of mycobacterium tuberculosis by using pcr and comparison it with ziehl neelsen staining and mantoux test. *Asian Journal of Phytomedicine and Clinical Research*. 1(4), 218-223
4. Al-Aghbari N., Al-Sonboli N., Yassin M.A., Coulter J.B., Atef Z., Al-Eryani A., Cuevas L.E. (2009). Multiple sampling in one day to optimize smear microscopy in children with tuberculosis in Yemen. *PLoS One*. 4(4), e5140.
5. Alcaide F., Benítez M.A., Escribà J.M., Martín R. (2000). Evaluation of the BACTEC MGIT 960 and the MB/BacT systems for recovery of mycobacteria from clinical specimens and for species identification byDNA AccuProbe. *J Clin Microbiol*. 38(1), 398-401.
6. Altindis M., Çetinkaya Z., Kalayci R. (2011). Detection of Mycobacterium isolates with different methods and their resistance ratios against anti-tuberculosis drugs. *J Clin Microbio*. 41, 1710-1711.
7. Anderson C., Inhaber N., Menzies D. (1995). Comparison of sputum induction with fiber-optic bronchoscopy in the diagnosis of tuberculosis. *Am J Respir Crit Care Med*. 152(5 Pt 1), 1570-4.
8. Armstrong A.R. (1951). The laryngeal swab specimen in the cultural diagnosis of pulmonary tuberculosis. *Can Med Assoc J*. 65(6), 575-8.
9. Ba F., Rieder H.L. (1999). A comparison of fluorescence microscopy with the Ziehl-Neelsen technique in the examination of sputum for acid-fast bacilli. *Int J Tuberc Lung Dis*. 3(12), 1101-5.
10. Balakrishna J., Shahapur P.R., Chakradhar P., Hussain Saheb S. (2013). Comparative Study of Different Staining Techniques-Ziehl neelsen Stain, Gabbet's Stain, Fluorochrome Stain for Detecting of Mycobacterium Tuberculosis in the Sputum. *J. Pharm. Sci. & Res*. 5(4), 89-92.
11. Baylan O., Kisa O., Albay A., Doganci L. (2004). Evaluation of a new automated, rapid, colorimetric culture system using solid medium for laboratory diagnosis of tuberculosis and determination of anti-tuberculosis drug susceptibility. *Int J Tuberc Lung Dis*. 8(6), 772-7.
12. Beck G.J., Nanda K. (1962). Use of superheated saline aerosols as a diagnostic measure in pulmonary tubercuosis. A preliminary report. *Dis Chest*. 42, 74-8.
13. Bell D.J., Dacombe R., Graham S.M., Hicks A., Cohen D., Chikaonda T., French N., Molyneux M.E., Zijlstra E.E., Squire S.B., Gordon S.B. (2009). Simple measures are

as effective as invasive techniques in the diagnosis of pulmonary tuberculosis in Malawi. *Int J Tuberc Lung Dis*. 13(1), 99-104.

14. Bennedsen J., Larsen S.O. (1966). Examination for tubercle bacili by fluorescence microscopy. *Scand J Respir Dis*. 47(2), 114-20.
15. Berggren Palme I., Gudetta B., Bruchfeld J., Eriksson M., Giesecke J. (2004). Detection of Mycobacterium tuberculosis in gastric aspirate and sputum collected from Ethiopian HIV-positive and HIV-negative children in a mixed in- and outpatient setting. *Acta Paediatr*. 93(3), 311-5.
16. Bhandari B., Singh S.V., Sharma V.K. (1971). Bacteriological diagnosis of pulmonary tuberculosis. A comparative study of gastric wash, laryngeal swab and lung puncture. *Indian J Pediatr*. 38(284), 349-53.
17. Bicmen C., Coskun M., Senol G. (2003). Comparison of Dio-TK and LJ media for primary culture of mycobacterium: a preliminary study for a new medium. *J Clin Microbio*. 41, 1710-1711.
18. Biswas S., Das A., Sinha A., Das S.K., Bairagya T.D. (2013). The role of induced sputum in the diagnosis of pulmonary tuberculosis. *Lung India*. 30(3), 199-202.
19. Boum Y., Orikiriza P., Rojas-Ponce G., Riera-Montes M., Atwine D., Nansumba M., Bazira J., Tuyakira E., De Beaudrap P., Bonnet M., Page A.L. (2013). Use of colorimetric culture methods for detection of Mycobacterium tuberculosis complex isolates from sputum samples in resource-limited settings. *J Clin Microbiol*. 51(7), 2273-9.
20. Brittle W., Marais B.J., Hesseling A.C., Schaaf H.S., Kidd M., Wasserman E., Botha T. (2009). Improvement in mycobacterial yield and reduced time to detection in pediatric samples by use of a nutrient broth growth supplement. *J Clin Microbiol*. 47(5), 1287-9.
21. Brown M., Varia H., Bassett P., Davidson R.N., Wall R., Pasvol G. (2007). Prospective study of sputum induction, gastric washing, and bronchoalveolar lavage for the diagnosis of pulmonary tuberculosis inpatients who are unable to expectorate. *Clin Infect Dis*. 44(11), 1415-20.
22. Brunello F., Favari F., Fontana R. (1999). Comparison of the MB/BacT and BACTEC 460 TB systems for recovery of mycobacteria from various clinical specimens. *J Clin Microbiol*. 37(4), 1206-9.
23. Cambau E., Wichlacz C., Truffot-Pernot C., Jarlier V. (1999). Evaluation of the new MB redox system for detection of growth of mycobacteria. *J Clin Microbiol*. 37(6), 2013-5.
24. Caviedes L., Lee T.S., Gilman R.H., Sheen P., Spellman E., Lee E.H., Berg D.E., Montenegro-James S. (2000). Rapid, efficient detection and drug susceptibility testing of Mycobacterium tuberculosis in sputum by microscopic observation of broth cultures. The Tuberculosis Working Group in Peru. *J Clin Microbiol*. 38(3), 1203-8.
25. Chan D.S., Choy M.Y., Wang S., Sng L.H. (2008). An evaluation of the recovery of mycobacteria from urine specimens using the automated Mycobacteria Growth Indicator Tube system (BACTEC MGIT 960). *J Med Microbiol*. 57(Pt 10), 1220-2.

26. Chihota V.N., Grant A.D., Fielding K., Ndibongo B., van Zyl A., Muirhead D., Churchyard G.J. (2010). Liquid vs. solid culture for tuberculosis: performance and cost in are source-constrained setting. *Int J Tuberc Lung Dis*. 14(8), 1024-31.
27. Coban A.Y., Akgüneş A., Durupınar B. (2011). Evaluation of blood agar medium for the growth of mycobacteria. *Mikrobiyol Bul*. 45(4), 617-22.
28. Dang T.M., Nguyen T.N., Wolbers M., Vo S.K., Hoang T.T., Nguyen H.D., To M.H., Vuong M.B., Nguyen T.P., Tran V.Q., Nguyen T.B., Vo T.H., Nguyen T.N., Dai V.H., Phan T.H., Nguyen H.D., Farrar J., Caws M. (2012). Evaluation of microscopic observation drug susceptibility assay for diagnosis of multidrug-resistant tuberculosis in Viet Nam. BMC Infect Dis. 1, 12:49.
29. Donald P.R., Schaaf H.S., Gie R.P., Beyers N., Sirgel F.A., Venter A. (1996). Stool microscopy and culture to assist the diagnosis of pulmonary tuberculosis in childhood. *J Trop Pediatr*. 42(5), 311-2.
30. Drancourt M., Raoult D. (2007). Cost-effectiveness of blood agar for isolation of mycobacteria. *PLoS Negl Trop Dis*. 1(2), e83.
31. Ercis S., Alp A., Hasçelik G., Kocagöz T. (2002). Comparison of Dio-TK rapid mycobacterium culture system with BACTEC 460 TB system and Löwenstein Jensen medium in diagnosis of tuberculosis and detection of susceptibility of antituberculosis drugs. Abant, Bolu, Turkey: 4th National Mycobacterium Symposium, 180–181.
32. Falconi F.Q., Suárez L.I., López Mde J., Sancho C.G. (2008). Comparison of the VersaTREK system and Löwenstein-Jensen medium for the recovery of mycobacteria from clinical specimens. *Scand J Infect Dis*. 40(1), 49-53.
33. Franchi L.M., Cama R.I., Gilman R.H., Montenegro-James S., Sheen P. (1998). Detection of Mycobacterium tuberculosis in nasopharyngeal aspirate samples in children. *Lancet*. 352(9141), 1681-2.
34. Gérôme P., Fabre M., Soler C.P., De Pina J.J., Simon F. (2009). Comparison of the mycobacteria growth indicator tube with solid culture for the detection of tuberculosis complex mycobacteria from blood. *Pathol Biol* (Paris). 57(1), 44-50.
35. Gil-Setas A., Torroba L., Fernandez J.L., Martinez-Artola V., Olite J. (2004). Evaluation of the MB/BacT system compared with Middlebrook 7H11 and Lowenstein-Jensen media for detection and recovery of mycobacteria from clinical specimens. *Clin Microbiol Infect*. 10(3), 224-8.
36. Githui W., Kitui F., Juma E.S., Obwana D.O., Mwai J., Kwamanga D. (1993). A comparative study on the reliability of the fluorescence microscopy and Ziehl-Neelsen method in the diagnosis of pulmonary tuberculosis. *East Afr Med J*. 70(5), 263-6.
37. Golia S., Hittinahalli V., Nirmala A.R., Sangeetha K.T., Asha S.K.B. (2013). A comparative study of auramine staining using led fluorescent microscopy with ziehl-neelsen staining in the diagnosis of pulmonary tuberculosis. *Journal of Evolution of Medical and Dental Sciences*. Volume 2, Issue 20.
38. Goyal R., Kumar A. (2013). A Comparison of Ziehl-Neelsen Staining and Fluorescent Microscopy for Diagnosis of Pulmonary TuberculosisIOSR Journal of Dental and Medical Sciences (IOSR-JDMS) e-ISSN: 2279-0853, p-ISSN: 2279-0861. Volume 8, Issue 5, PP 05-08.

39. Habtamu M., van den Boogaard J., Ndaro A., Buretta R., Irongo C.F., Lega D.A., Nyombi B.M., Kibiki G.S. (2012). Light-emitting diode with various sputum smear preparation techniques to diagnose tuberculosis. *Int J Tuberc Lung Dis*. 16(3), 402-7.
40. Hanna B.A., Ebrahimzadeh A., Elliott L.B., Morgan M.A., Novak S.M., Rusch-Gerdes S., Acio M., Dunbar D.F., Holmes T.M., Rexer C.H., Savthyakumar C., Vannier A.M. (1999). Multicenter evaluation of the BACTEC MGIT 960 system for recovery of mycobacteria. *J Clin Microbiol*. 37(3), 748-52.
41. Harris G., Rayner A., Blair J., Watt B. (2000). Comparison of three isolation systems for the culture of mycobacteria from respiratory and non-respiratory samples. *J Clin Pathol*. 53(8), 615-8.
42. Heifets L., Linder T., Sanchez T., Spencer D., Brennan J. (2000). Two liquid medium systems, mycobacteria growth indicator tube and MB redox tube, for Mycobacterium tuberculosis isolation from sputum specimens. *J Clin Microbiol*. 38(3), 1227-30.
43. Hensler N.M., Spivey C.G. Jr, Dees T.M. (1961). The use of hypertonic aerosol in production of sputum for diagnosis oftuberculosis. Comparison with gastric specimens. *Dis Chest*. 40, 639-42.
44. Hillemann D., Richter E., Rüsch-Gerdes S. (2006). Use of the BACTEC Mycobacteria Growth Indicator Tube 960 automated system for recovery of Mycobacteria from 9,558 extrapulmonary specimens, including urine samples. *J Clin Microbiol*. 44(11), 4014-7.
45. Hooja S., Pal N., Malhotra B., Goyal S., Kumar V., Vyas L. (2011). Comparison of Ziehl Neelsen & Auramine O staining methods on direct and concentrated smears in clinical specimens. *Indian J Tuberc*. 58(2), 72-6.
46. Huang T.S., Chen C.S., Lee S.S., Huang W.K., Liu Y.C. (2001). Comparison of the BACTEC MGIT 960 and BACTEC 460TB systems for detection of mycobacteria in clinical specimens. *Ann Clin Lab Sci*. 31(3), 279-83.
47. Idigoras P., Beristain X., Iturzaeta A., Vicente D., Pérez-Trallero E. (2000). Comparison of the automated nonradiometric Bactec MGIT 960 system with Löwenstein-Jensen, Coletsos, and Middlebrook 7H11 solid media for recovery of mycobacteria. *Eur J Clin Microbiol Infect Dis*. 19(5), 350-4.
48. Jain A., Bhargava A., Agarwal S.K. (2002). A comparative study of two commonly used staining techniques for acid fast bacilli In clinical specimens. *Ind. J Tub*. 49, 161.
49. Jones F.L. Jr. (1966). The relative efficacy of spontaneous sputa, aerosol-induced sputa, and gastric aspirates in the bacteriologic diagnosis of pulmonary tuberculosis. *Dis Chest*. 50(4), 403-8.
50. Kanchana M.V., Cheke D., Natyshak I., Connor B., Warner A., Martin T. (2000). Evaluation of the BACTEC MGIT 960 system for the recovery of mycobacteria. Diagn Microbiol Infect Dis. 37(1), 31-6.
51. Kaufmann S.H.E., Hahn H. (2003). Mycobacteria and TB: Issues in infectious diseases. Editors: H. Zeichhardt, B. Mahy. Vol.2.
52. Khazaei S., Izadi B., Zandieh Z., Alvandimanesh A., Vaziri S. (2014). Comparison of Polymerase Chain Reaction, Ziehl-Neelsen Staining and Histopathologic Findings in Formalin-fixed, Paraffin-Embedded Tissue Specimens for Diagnosis of Tuberculosis. *Iranian Journal of Pathology*. 9(3), 208 - 214.

53. Kivihya-Ndugga L., van Cleeff M., Juma E., Kimwomi J., Githui W., Oskam L., Schuitema A., van Soolingen D., Nganga L., Kibuga D., Odhiambo J., Klatser P. (2004). Comparison of PCR with the routine procedure for diagnosis of tuberculosis in a population with highprevalences of tuberculosis and human immunodeficiency virus. *J Clin Microbiol*. 42(3), 1012-5.
54. Kivihya-Ndugga L.E., van Cleeff M.R., Githui W.A., Nganga L.W., Kibuga D.K., Odhiambo J.A., Klatser P.R. (2003). A comprehensive comparison of Ziehl-Neelsen and fluorescence microscopy for the diagnosis of tuberculosis in a resource-poor urban setting. *Int J Tuberc Lung Dis*. 7(12), 1163-71.
55. Kiwanuka J., Graham S.M., Coulter J.B., Gondwe J.S., Chilewani N., Carty H., Hart C.A. (2001). Diagnosis of pulmonary tuberculosis in children in an HIV-endemic area, Malawi. *Ann Trop Paediatr*. 21(1), 5-14.
56. Kocagoz T., Alp A., Albay A. (2000). A new rapid non-radioactive medium for culturing mycobacteria, that also enables visually differentiation of mycobacterial growth from contamination. *J Clin Microbio*. 41, 1710-1711.
57. Kocagöz T., Altın S., Türkyılmaz Ö., Taş İ., Karaduman P., Bolaban D., Yeşilyurt E., Öktem S., Aytekin N., Şınık G., Mozioğlu E., Silier T. (2012). Efficiency of the TK Culture System in the diagnosis of tuberculosis. *Diagn Microbiol Infect Dis*. 72(4), 350-7.
58. Kumar P., Sen M.K., Chauhan D.S., Katoch V.M., Singh S., Prasad H.K. (2010). Assessment of the N PCR assay in diagnosis of pleural tuberculosis: detection of M. tuberculosis in pleural fluid and sputum collected in tandem. *PLoS One*. 5(4), e10220.
59. Kumudini T., Shrihari N. (2012). A Comparison of three different staining methods for the detection of acid fast bacilli (Mycobacterium tuberculosis) in sputum samples. *JPBMS*. 14(06).
60. Laifangbam S., Singh H.L., Singh N.B., Devi K.M., Singh N.T. (2009). A comparative study of fluorescent microscopy with Ziehl-Neelsen staining and culture for the diagnosis of pulmonary tuberculosis. *Kathmandu University Medical Journal*. 27, 226-230.
61. Lazarus R.P., Kalaiselvan S., John K.R., Michael J.S. (2012). Evaluation of the microscopic observational drug susceptibility assay for rapid and efficient diagnosis of multi-drug resistant tuberculosis. *Indian J Med Microbiol*. 30(1), 64-8.
62. Leitritz L., Schubert S., Bücherl B., Masch A., Heesemann J., Roggenkamp A. (2001). Evaluation of BACTEC MGIT 960 and BACTEC 460TB systems for recovery of mycobacteria from clinical specimens of a university hospital with low incidence of tuberculosis. *J Clin Microbiol*. 39(10), 3764-7.
63. Levidiotou S., Papamichael D., Gessouli E., Golegou S., Anagnostou S., Galanakis E., Papadopoulou C., Antoniadis G. (1999). Detection of mycobacteria in clinical specimen using the mycobacteria growth indicator tube (MGIT) and the Lowenstein Jensen medium. *Microbiol Res*. 154(2), 151-5.
64. Lloyd A.V. (1968). Bacteriological diagnosis of tuberculosis in children: a comparative study of gastric lavage and laryngeal swab methods. *East Afr Med J*. 45(3),140-3.

65. Lobato M.N., Loeffler A.M., Furst K., Cole B., Hopewell P.C. (1998). Detection of Mycobacterium tuberculosis in gastric aspirates collected from children: hospitalization is not necessary. *Pediatrics*. 102(4), E40.
66. Lu D., Heeren B., Dunne W.M. (2002). Comparison of the Automated Mycobacteria Growth Indicator Tube System (BACTEC960/MGIT) with Löwenstein Jensen medium f or recovery of mycobacteria from clinical specimens. *Am J Clin Pathol*. 118(4), 542-5.
67. Macondo E.A., Ba F., Toure-Kane N.C., Kaire O., Gueye-Ndiaye A., Gaye-Diallo A., Boye C.S., Mboup S. (2000). Improvement of tuberculosis diagnosis by the Mycobacteria Growth Indicator Tube (MGIT) in a developing country laboratory. *Bull Soc Pathol Exot*. 93(2), 97-100.
68. Makamure B., Mhaka J., Makumbirofa S., Mutetwa R., Mupfumi L., Mason P., Metcalfe J.Z. (2013). Microscopic-observation drug-susceptibility assay for the diagnosis of drug-resistant tuberculosis in Harare, Zimbabwe. *PLoS One*. 8(2), e55872.
69. Mankiewicz E. (1953). Modified laryngeal swab method for the detection of tubercle bacilli in pulmonary tuberculosis. *Can Med Assoc J*. 69(2), 160-1.
70. Marais B.J., Brittle W., Painczyk K., Hesseling A.C., Beyers N., Wasserman E., van Soolingen D., Warren R.M. (2008). Use of light-emitting diode fluorescence microscopy to detect acid-fast bacilli in sputum. *Clin Infect Dis*. 47(2), 203-7.
71. Martin A., Munga Waweru P., Babu Okatch F., Amondi Ouma N., Bonte L., Varaine F., Portaels F. (2009). Implementation of the thin layer agar method for diagnosis of smear-negative pulmonary tuberculosis in a setting with a high prevalence of human immunodeficiency virus infection in Homa Bay, Kenya. *J Clin Microbiol*. 47(8), 2632-4.
72. Marzouk M., Ferjani A., Dhaou M., Ali M.H., Hannachi N., Boukadida J. (2013). Comparison of LED and conventional fluorescence microscopy for detection of acid-fast bacilli in an area with high tuberculosis incidence. Diagn Microbiol Infect Dis. 76(3), 306-8.
73. Mathur M.L., Gaur J., Sharma R., Solanki A. (2009). Rapid culture of Mycobacterium tuberculosis on blood agar in resource limited setting. *Dan Med Bull*. 56(4), 208-10.
74. McWilliams T., Wells A.U., Harrison A.C., Lindstrom S., Cameron R.J., Foskin E. (2002). Induced sputum and bronchoscopy in the diagnosis of pulmonary tuberculosis. *Thorax*. 57(12), 1010-4.
75. Mejia G.I., Castrillon L., Trujillo H., Robledo J.A. (1999). Microcolony detection in 7H11 thin layer culture is an alternative for rapid diagnosis of Mycobacteriumtuberculosis infection. *Int J Tuberc Lung Dis*. 3(2), 138-42.
76. Migliori G.B., Borghesi A., Rossanigo P., Adriko C., Neri M., Santini S., Bartoloni A., Paradisi F., Acocella G. (1992). Proposal of an improved score method for the diagnosis of pulmonary tuberculosis in childhood in developing countries. *Tuber Lung Dis*. 73(3), 145-9.
77. Mirovic V., Lepsanovic Z. (2002). Evaluation of the MB/BacT system for recovery of mycobacteria from clinical specimens incomparison to Lowenstein-Jensen medium. *Clin Microbiol Infect*. 8(11), 709-14

78. Moore D.A., Mendoza D., Gilman R.H., Evans C.A., Hollm Delgado M.G., Guerra J., Caviedes L., Vargas D, Ticona E., Ortiz J., Soto G., Serpa J., Tuberculosis Working Group in Peru. (2004). Microscopic observation drug susceptibility assay, a rapid, reliable diagnostic test for multidrug-resistant tuberculosis suitable for use in resource-poor settings. *J Clin Microbiol*. 42(10), 4432-7.
79. Moreira Ada S., Huf G., Vieira M.A., Fonseca L., Ricks M., Kritski A.L. (2013). Performance comparison between the mycobacteria growth indicator tube system and Löwenstein-Jensen medium in the routine detection of Mycobacterium tuberculosis at public health care facilities in Rio de Janeiro, Brazil: preliminary results of a pragmatic clinical trial. *J Bras Pneumol*. 39(3), 365-7.
80. Moreno R., García del Busto A., Pardo F., Galiano J.V., Sabater S., Hernández I. (1999). Evaluation of an automatic system (MB/BacT) for the isolation of Mycobacterium spp. *Enferm Infecc Microbiol Clin*. 17(3), 126-9.
81. Morse M., Kessler J., Albrecht S., Kim R., Thakur R., Nthobatsang R., Radisowa K., Maunatlala C., Yang W., Macgregor R.R., Friedman H. (2008). Induced sputum improves the diagnosis of pulmonary tuberculosis in hospitalized patients in Gaborone, Botswana. *Int J Tuberc Lung Dis*. 12(11), 1279-85.
82. Muyoyeta M., Schaap J.A., De Haas P., Mwanza W., Muvwimi M.W., Godfrey-Faussett P., Ayles H. (2009). Comparison of four culture systems for Mycobacterium tuberculosis in the Zambian National Reference Laboratory. *Int J Tuberc Lung Dis*. 13(4), 460-5.
83. Naveen G., Peerapur B.V. (2012). Comparison of the Lowenstein-Jensen Medium, the Middlebrook 7H10Medium and MB/BacT for the Isolation of Mycobacterium Tuberculosis(MTB) from Clinical Specimens. *J Clin Diagn Res*. 6(10), 1704-9.
84. Norrman E., Keistinen T., Uddenfeldt M., Rydström P.O., Lundgren R. (1988). Bronchoalveolar lavage is better than gastric lavage in the diagnosis of pulmonary tuberculosis. *Scand J Infect Dis*. 20(1), 77-80.
85. Oberhelman R.A., Soto-Castellares G., Caviedes L., Castillo M.E., Kissinger P., Moore D.A., Evans C., Gilman R.H. (2006). Improved recovery of Mycobacterium tuberculosis from children using the microscopic observation drug susceptibility method. *Pediatrics*. 118(1), e100-6.
86. Oberhelman R.A., Soto-Castellares G., Gilman R.H., Caviedes L., Castillo M.E., Kolevic L., Del Pino T., Saito M., Salazar-Lindo E., Negron E., Montenegro S., Laguna-Torres V.A., Moore D.A., Evans C.A. (2010). Diagnostic approaches for paediatric tuberculosis by use of different specimen types, culture methods, and PCR: a prospective case-control study. *Lancet Infect Dis*. 10(9), 612-20.
87. Otu J., Antonio M., Cheung Y.B., Donkor S., De Jong B.C., Corrah T., Adegbola R.A. (2008). Comparative evaluation of BACTEC MGIT 960 with BACTEC 9000 MB and LJ for isolation of mycobacteria in The Gambia. *J Infect Dev Ctries*. 2(3), 200-5.
88. Owens S., Abdel-Rahman I.E., Balyejusa S., Musoke P., Cooke R.P., Parry C.M., Coulter J.B. (2007). Nasopharyngeal aspiration for diagnosis of pulmonary tuberculosis. *Arch Dis Child*. 92(8), 693-6.
89. Palacios J.J., Ferro J., Ruiz Palma N., García J.M., Villar H., Rodríguez J., Macías M.D., Prendes P. (1999). Fully automated liquid culture system compared with

Löwenstein-Jensen solid medium for rapid recovery of mycobacteria from clinical samples. *Eur J Clin Microbiol Infect Dis*. 18(4), 265-73.

90. Panicker J.N., Nagaraja D., Subbakrishna D.K., Venkataswamy M.M., Chandramuki A. (2010). Role of the BACTEC radiometric method in the evaluation of patients with clinically probable tuberculous meningitis. *Ann Indian Acad Neurol*. 13(2), 128-31.
91. Petrović S. (2005). Diagnostic value of certain methods for isolation of Mycobacteriumtuberculosis in children with suspected pulmonary tuberculosis. *Med Pregl*. 58(5-6), 231-5.
92. Piersimoni C., Scarparo C., Callegaro A., Tosi C.P., Nista D., Bornigia S., Scagnelli M., Rigon A., Ruggiero G., Goglio A. (2001). Comparison of MB/Bact alert 3D system with radiometric BACTEC system and Löwenstein-Jensen medium for recovery and identification of mycobacteria from clinical specimens: a multicenter study. *J Clin Microbiol*. 39(2), 651-7.
93. Piersimoni C., Scarparo C., Cichero P., De Pezzo M., Covelli I., Gesu G., Nista D., Scagnelli M., Mandler F. (1999). Multicenter evaluation of the MB-Redox medium compared with radiometric BACTEC system, mycobacteria growth indicator tube (MGIT), and Löwenstein-Jensen medium for detection and recovery of acid-fast bacilli. *Diagn Microbiol Infect Dis*.34(4), 293-9.
94. Pinheiro M.D., Ribeiro M.M. (2000). Comparison of the Bactec 460TB system and the Bactec MGIT 960 system in recovery of mycobacteria fromclinical specimens. *Clin Microbiol Infect*. 6(3), 171-3.
95. Prasanthi K., Kumari A.R. (2005). Efficacy of fluorochrome stain in the diagnosis of pulmonary tuberculosis co-infected with HIV. *Indian J Med Microbiol*. 23(3), 179-81.
96. Ramarokoto H., Rasolonavalona T., Ratsimba L., Andrianasolo D., Ratsitorahina M., Rasolofo Razanamparany V. (2007). Evaluation of a rapid culture method on liquid Bio FM (BIO-RAD) medium for the isolation of mycobacteria. *Int J Tuberc Lung Dis*. 11(8), 898-903.
97. Robledo J.A., Mejía G.I., Morcillo N., Chacón L., Camacho M., Luna J., Zurita J., Bodon A., Velasco M., Palomino J.C., Martin A., Portaels F. (2006). Evaluation of a rapid culture method for tuberculosis diagnosis: a Latin American multi-center study. *Int J Tuberc Lung Dis*. 10(6), 613-9.
98. Roggenkamp A., Hornef M.W., Masch A., Aigner B., Autenrieth I.B., Heesemann J. (1999). Comparison of MB/BacT and BACTEC 460 TB systems for recovery of mycobacteria in a routine diagnostic laboratory. *J Clin Microbiol*. 37(11), 3711-2.
99. Rohner P., Ninet B., Benri A.M., Auckenthaler R. (2000). Evaluation of the Bactec 960 automated nonradiometric system for isolation of mycobacteria from clinical specimens. *Eur J Clin Microbiol Infect Dis*. 19(9), 715-7.
100. Ruiz Jiménez M., Guillén Martín S., Prieto Tato L.M., Cacho Calvo J.B., Álvarez García A., Soto Sánchez B., Ramos Amador J.T. (2013). "Induced sputum versus gastric lavage for the diagnosis of pulmonary tuberculosis in children". *BMC Infect Dis*. 16, 13:222.
101. Runa F., Yasmin M., Hoq M.M., Begum J., Rahman A.S., Ahsan C.R. (2011). Molecular versus conventional methods: clinical evaluation of different methods for thediagnosis of tuberculosis in Bangladesh. *J Microbiol Immunol Infect*. 44(2), 101-5.

102. Saglam L., Akgun M., Aktas E. (2005). Usefulness of induced sputum and fibreoptic bronchoscopy specimens in the diagnosis of pulmonary tuberculosis. J Int Med Res. 33(2), 260-5.

103. Saleh M.A.D., Khaleel K.J., Salman Alsaadi L.A. (2014). Comparison between polymerase chain reaction and Ziehl Neelsen stain for detection renal tuberculosis. *Int.J.Curr.Microbiol.App.Sci*. 3(6), 408-414.

104. Samra Z., Kaufman L., Bechor J., Bahar J. (2000). Comparative study of three culture systems for optimal recovery of mycobacteria from different clinical specimens. *Eur J Clin Microbiol Infect Dis*. 19(10), 750-4.

105. Satti L., Ikram A., Abbasi S., Malik N., Mirza I.A., Martin A. (2010). Evaluation of thin-layer agar 7H11 for the isolation of Mycobacterium tuberculosis complex. *Int J Tuberc Lung Dis*. 14(10), 1354-6.

106. Sawadogo T.L., Savadogo L.G., Diande S., Ouedraogo F, Mourfou A, Gueye A, Sawadogo I, Nebié B, Sangare L, Ouattara AS. (2012). Comparison of Kinyoun, auramine O, and Ziehl-Neelsen staining for diagnosing tuberculosis at the National Tuberculosis Center in Burkina Faso. Med Sante Trop. 22(3), 302-6.

107. Saxena P., Asthana A.K., Madan M. (2014). Comparison of microscopy and PCR in detection of Mycobacterium tuberculosis. *JMID*. 4(4), 141-144.

108. Scarparo C., Piccoli P., Rigon A., Ruggiero G., Ricordi P., Piersimoni C. (2002). Evaluation of the BACTEC MGIT 960 in comparison with BACTEC 460 TB for detection and recovery of mycobacteria from clinical specimens. *Diagn Microbiol Infect Dis*. 44(2), 157-61.

109. Schoch O.D., Rieder P., Tueller C., Altpeter E., Zellweger J.P., Rieder H.L., Krause M., Thurnheer R. (2007). Diagnostic yield of sputum, induced sputum, and bronchoscopy after radiologic tuberculosis screening. *Am J Respir Crit Care Med*. 175(1), 80-6.

110. Sharp S.E., Lemes M., Sierra S.G., Poniecka A., Poppiti R.J. Jr. (2000). Löwenstein-Jensen media. No longer necessary for mycobacterial isolation. *Am J Clin Pathol*. 113(6), 770-3.

111. Siena I., Adi K., Gernowo R., Mirnasari N. (2012). Development of Algorithm Tuberculosis Bacteria Identification Using Color Segmentation and Neural Networks. *IJVIPNS-IJENS*. Vol:12 No:04.

112. Singh M., Moosa N.V., Kumar L., Sharma M. (2000). Role of gastric lavage and broncho-alveolar lavage in the bacteriological diagnosis of childhood pulmonary tuberculosis. *Indian Pediatr*. 37(9), 947-51.

113. Somoskövi A., Ködmön C., Lantos A., Bártfai Z., Tamási L., Füzy J., Magyar P. (2000). Comparison of recoveries of mycobacterium tuberculosis using the automated BACTEC MGIT 960 system, the BACTEC 460 TB system, and Löwenstein-Jensen medium. *J Clin Microbiol*. 38(6), 2395-7.

114. Somoskövi A., Ködmön C., Lantos A., Bártfai Z., Tamási L., Füzy J., Magyar P. (2000). Comparison of recoveries of mycobacterium tuberculosis using the automated BACTEC MGIT 960 system, the BACTEC 460 TB system, and Löwenstein-Jensen medium. *J Clin Microbiol*. 38(6), 2395-7.

115. Somoskövi A., Magyar P. (1999). Comparison of the mycobacteria growth indicator tube with MB redox, Löwenstein-Jensen, and Middlebrook 7H11 media for recovery of mycobacteria in clinical specimens. *J Clin Microbiol*. 37(5), 1366-9.
116. Somu N., Swaminathan S., Paramasivan C.N., Vijayasekaran D., Chandrabhooshanam A., Vijayan V.K., Prabhakar R. (1995). Value of bronchoalveolar lavage and gastric lavage in the diagnosis of pulmonary tuberculosis in children. *Tuber Lung Dis*. 76(4), 295-9.
117. Sorlozano A., Soria I., Roman J., Huertas P., Soto M.J., Piedrola G., Gutierrez J. (2009). Comparative evaluation of three culture methods for the isolation of mycobacteria from clinical samples. *J Microbiol Biotechnol*. 19(10), 1259-64.
118. Srisuwanvilai L.O., Monkongdee P., Podewils L.J., Ngamlert K., Pobkeeree V., Puripokai P., Kanjanamongkolsiri P., Subhachaturas W., Akarasewi P., Wells C.D., Tappero J.W., Varma J.K. (2008). Performance of the BACTEC MGIT 960 compared with solid media for detection of Mycobacterium in Bangkok, Thailand. *Diagn Microbiol Infect Dis*. 61(4), 402-7.
119. Tansuphasiri U., Kladphuang B. (2002). Evaluation of sputum staining by modified cold method and comparison with Ziehl Neelsen and fluorochrome methods for the primary diagnosis of tuberculosis. *Southeast Asian J Trop Med Public Health*. 33(1), 128-35.
120. Thakur R., Goyal R., Sarma S. (2010). Laboratory diagnosis of tuberculous meningitis - is there a scope for further improvement? *J Lab Physicians*. 2(1), 21-4.
121. Tortoli E., Cichero P., Piersimoni C., Simonetti M.T., Gesu G., Nista D. (1999). Use of BACTEC MGIT 960 for recovery of mycobacteria from clinical specimens: multicenter study. *J Clin Microbiol*. 37(11), 3578-82.
122. Tovar M., Siedner M.J., Gilman R.H., Santillan C., Caviedes L., Valencia T., Jave O., Escombe A.R., Moore D.A., Evans C.A. (2008). Improved diagnosis of pleural tuberculosis using the microscopic- observation drug-susceptibility technique. *Clin Infect Dis*. 46(6), 909-12.
123. Trusov A., Bumgarner R., Valijev R., Chestnova R., Talevski S., Vragoterova C., Neeley E.S. (2009). Comparison of Lumin LED fluorescent attachment, fluorescent microscopy and Ziehl-Neelsen for AFB diagnosis. Int J Tuberc Lung Dis. 13(7), 836-41.
124. Ulukanligil M., Aslan G., Tasçi S. (2000). A comparative study on the different staining methods and number of specimens for the detection of acid fast bacilli. *Mem Inst Oswaldo Cruz*. 95(6), 855-8.
125. van Cleeff M., Kivihya-Ndugga L., Githui W., Ng'ang'a L., Kibuga D., Odhiambo J., Klatser P. (2005). Cost-effectiveness of polymerase chain reaction versus Ziehl-Neelsen smear microscopy for diagnosis of tuberculosis in Kenya. *Int J Tuberc Lung Dis*. 9(8), 877-83.
126. Vargas D., García L., Gilman R.H., Evans C., Ticona E., Navincopa M., Luo R.F., Caviedes L., Hong C., Escombe R., Moore D.A. (2005). Diagnosis of sputum-scarce HIV-associated pulmonary tuberculosis in Lima, Peru. *Lancet*. 365(9454), 150-2.

127. Whyte T., Cormican M., Hanahoe B., Doran G., Collins T., Corbett-Feeney G. (2000). Comparison of BACTEC MGIT 960 and BACTEC 460 for culture of Mycobacteria. *Diagn Microbiol Infect Dis*. 38(2), 123-6.
128. Williams-Bouyer N., Yorke R., Lee H.I., Woods G.L. (2000). Comparison of the BACTEC MGIT 960 and ESP culture system II for growth and detection of mycobacteria. *J Clin Microbiol*. 38(11), 4167-70.
129. Xia H., Song Y.Y., Zhao B., Kam K.M., O'Brien R.J., Zhang Z.Y., Sohn H., Wang W., Zhao Y.L. (2013). Multicentre evaluation of Ziehl-Neelsen and light-emitting diode fluorescence microscopyin China. Int J Tuberc *Lung Dis*. 17(1), 107-12.
130. Yan J.J., Huang A.H., Tsai S.H., Ko W.C., Jin Y.T., Wu J.J. (2000). Comparison of the MB/BacT and BACTEC MGIT 960 system for recovery of mycobacteria from clinical specimens. Diagn Microbiol Infect Dis. 37(1), 25-30.
131. Yue W.Y., Cohen S.S. (1967). Sputum induction by newer inhalation methods in patients with pulmonary tuberculosis. *Dis Chest*. 51(6), 614-20.
132. Zar H.J., Hanslo D., Apolles P., Swingler G., Hussey G. (2005). Induced sputum versus gastric lavage for microbiological confirmation of pulmonary tuberculosis in infants and young children: a prospective study. *Lancet*. 365(9454), 130-4.
133. Zar H.J., Tannenbaum E., Apolles P., Roux P., Hanslo D., Hussey G. (2000). Sputum induction for the diagnosis of pulmonary tuberculosis in infants and young children in an urban setting in South Africa. *Arch Dis Child*. 82(4), 305-8.
134. Zar H.J., Tannenbaum E., Hanslo D., Hussey G. (2003). Sputum induction as a diagnostic tool for community-acquired pneumonia in infants and young children from a high HIV prevalence area. *Pediatr Pulmonol*. 36(1), 58-62.
135. Zar H.J., Workman L., Isaacs W., Munro J., Black F., Eley B., Allen V., Boehme C.C., Zemanay W., Nicol M.P. (2012). Rapid molecular diagnosis of pulmonary tuberculosis in children using nasopharyngeal specimens. *Clin Infect Dis*. 55(8), 1088-95.
